# Update on the Protein Homeostasis Network in *Bacillus subtilis*

**DOI:** 10.3389/fmicb.2022.865141

**Published:** 2022-03-08

**Authors:** Judith Matavacas, Claes von Wachenfeldt

**Affiliations:** Department of Biology, Lund University, Lund, Sweden

**Keywords:** chaperone, protease, degradation tags, protein quality control, protein aggregation, proteotoxic stress

## Abstract

Protein homeostasis is fundamental to cell function and survival. It relies on an interconnected network of processes involving protein synthesis, folding, post-translational modification and degradation as well as regulators of these processes. Here we provide an update on the roles, regulation and subcellular localization of the protein homeostasis machinery in the Gram-positive model organism *Bacillus subtilis*. We discuss emerging ideas and current research gaps in the field that, if tackled, increase our understanding of how Gram-positive bacteria, including several human pathogens, maintain protein homeostasis and cope with stressful conditions that challenge their survival.

## Introduction

**FIGURE 1:**
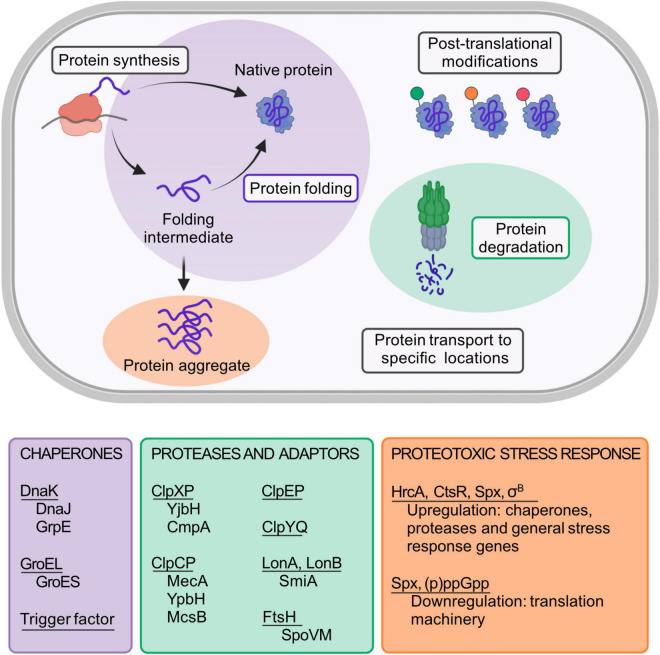
Schematic representation of the *B. subtilis* proteostasis network and its regulators. Protein folding and degradation are two key cellular processes involved in proteostasis maintenance. While some proteins can spontaneously fold into their functional native state, many others need the assistance of molecular chaperones to do so. Underlined in the purple box are the main *B. subtilis* chaperones. For its full activity, DnaK requires the co-chaperone DnaJ and the nucleotide exchange factor GrpE. Similarly, GroEL requires the co-chaperonin GroES. Unneeded, misfolded or damaged proteins are eliminated from the cell by proteases (underlined in the green box) and its respective adaptor proteins (shown below each protease complex). In addition to protein folding and degradation, other key processes that affect proteostasis maintenance are protein synthesis, post-translational modifications, and the transport of proteins to specific locations. Failure in proteostasis maintenance often leads to the formation and accumulation of misfolded and aggregated proteins, a condition termed as proteotoxic stress. The main regulators that are known to be involved in the *B. subtilis* proteotoxic stress response are shown in the orange text box. Created with BioRender.com.

Native proteins typically fold into well-defined three-dimensional structures. To function properly, all cells need to contain correctly folded proteins and have mechanisms to prevent accumulation of unneeded or aberrant proteins. The folded state of most proteins is marginally more stable than the unfolded state. Therefore, small changes of environmental conditions may affect the equilibrium between the folded and unfolded state. Protein homeostasis (proteostasis) is crucial to achieve a “healthy” proteome, and refers to the dynamic balance between synthesis, folding, post-translational modification, transport, and degradation of proteins (Figure 1; Powers et al., 2009; Richter et al., 2010; Schramm et al., 2020). The main components of the proteostasis network are the ancient and evolutionary conserved chaperones and proteases, which assist in protein folding and degrade specific protein substrates, respectively (Powers and Balch, 2013; Balchin et al., 2016; Olivares et al., 2016). Surprisingly, even though eukaryotic proteomes are typically much larger and complex and contain more aggregation-prone proteins than those of prokaryotes, no new core chaperones appear to have emerged during billion years of evolution (Rebeaud et al., 2021). Instead, the core chaperones and their relative abundance have remained invariant across the domains of life. Maintaining integrity of a more complex and unstable proteome has been dealt with by increasing cellular chaperone levels, as well as promoting cooperation between them (Rebeaud et al., 2021).

Understanding how cells maintain proteostasis is an important topic to address, not only because proteome integrity is crucial for the correct cellular function, but also because accumulation of protein aggregates – which mainly results from dysregulation of proteostasis – has been linked to aging and to human diseases, such as Parkinson’s and Alzheimer’s, and to defects in growth and survival in prokaryotes (Balchin et al., 2016; Cheng et al., 2018). In addition, the presence of protein aggregates is strongly correlated with dormant antibiotic-resistant cells, called persisters (Leszczynska et al., 2013; Pu et al., 2019; Yu et al., 2019; Dewachter et al., 2021; Huemer et al., 2021). Because of this and other data, disruption of proteostasis has been suggested as an anti-bacterial strategy (Khodaparast et al., 2021).

Most information on chaperones and proteases in bacteria derives from studies in *Escherichia coli*, but there are many host-specific regulation mechanisms concerning proteostasis to unravel. *B. subtilis* is adapted to rapid intracellular and environmental fluctuations that challenge the stability of its proteome. Thus, it is a suitable model organism to study not only from the view of fundamental principles of proteostasis, but also regarding proteostasis maintenance in other Gram-positive bacteria, that includes several human pathogens. Here we provide an updated description of the main components of the *B. subtilis* proteostasis network (Figure 1), and address known and emerging mechanisms for its regulation during proteotoxic stress.

## The Major Classes of Molecular Chaperones: Conserved Mechanisms of Action and Roles in *B. subtilis*

Molecular chaperones are central to proteostasis by ensuring that proteins are correctly folded, and preventing protein misfolding and aggregation (Mogk et al., 2011; Balchin et al., 2016). The ancient and evolutionary conserved DnaK (Hsp70), GroEL (Hsp60), and trigger factor (TF) are three important abundant cytosolic chaperones in *B. subtilis* (Moliere and Turgay, 2009).

DnaK functions as a monomer and consists of an N-terminal ATPase domain and a C-terminal peptide-binding domain composed of a β-sandwich and an α-helical lid (Zhu et al., 1996; Perales-Calvo et al., 2018). Together with its co-chaperone DnaJ (Hsp40) and the nucleotide exchange factor GrpE, and through ATP hydrolysis, the α-helical lid closes over the β-sandwich, allowing tight binding of unfolded substrates (Liberek et al., 1991; Zhu et al., 1996). DnaK typically recognizes exposed hydrophobic peptide segments (∼5–7 residues) of client proteins that are prone to aggregate during folding (Rudiger et al., 1997; Mogk et al., 1999; Calloni et al., 2012); substrate binding and release cycles decrease the folding rate, and prevent non-native protein species from folding prematurely in a misfolded state or from aggregating (Szabo et al., 1994). Interestingly, some bacteria like *E. coli* contain more than one Hsp70 and Hsp40 homolog. For instance, apart from DnaK, *E. coli* harbors two additional Hsp70 proteins – Hsc66 (Seaton and Vickery, 1994) and Hsc62 (Yoshimune et al., 1998) – and five additional DnaJ-like proteins such as Hsc20 and CbpA (Ueguchi et al., 1994; Lelivelt and Kawula, 1995; Clarke et al., 1996; Itoh et al., 1999; Yoshimune et al., 2002). In contrast, *B. subtilis* appears to have only DnaK and DnaJ. Oligomeric GroEL is composed of two stacked heptameric rings, each forming large cylindrical cavities in which misfolded protein substrates can be enclosed (Langer et al., 1992; Mayhew et al., 1996). The GroES (Hsp10) heptameric co-chaperonin caps GroEL cavities, and through ATP hydrolysis allows complete substrate encapsulation, thus providing a “protected” folding environment (Mayhew et al., 1996). Finally, TF is a ribosome-associated chaperone comprised of three domains adopting an overall elongated shape (Stoller et al., 1995; Zarnt et al., 1997). An N-terminal ribosome-binding domain is followed by a peptidyl-prolyl isomerase domain linked to the C-terminal substrate-binding domain. The substrate-binding domain has two helical arms that form a promiscuous clamp-like structure, providing a shielded environment to nascent polypeptides as translation proceeds, and also slowing the folding rate preventing aggregation (Agashe et al., 2004; Ferbitz et al., 2004; Singhal et al., 2015). The clamp, together with the TF structural flexibility, allows TF to function with a wide range of emerging substrates (Martinez-Hackert and Hendrickson, 2009; Saio et al., 2014). While the monomeric form of TF is bound to the ribosome, its dimeric form exists mainly in the cytosol, and apart from assisting several proteins in their folding, it has anti-aggregation activity (Saio et al., 2018).

In addition to assisting in the folding of cytosolic proteins, DnaK/DnaJ/GrpE (Wild et al., 1992, 1993, 1996), GroEL/GroES (Kusukawa et al., 1989), and TF (Lee and Bernstein, 2002; Genevaux et al., 2004; Ullers et al., 2007; Oh et al., 2011) are also involved in protein secretion by preventing premature folding and aggregation of presecretory proteins in the cytosol. Most proteins are translocated in an unfolded state via the general secretion (Sec) pathway. Typically, the secretion-specific chaperone SecB binds newly synthesized presecretory proteins and targets them for SecA-driven protein translocation. However, SecB is absent in many Gram-positive bacteria, and in *B. subtilis* CsaA has been suggested to play a similar role since, among other evidence, it interacts with SecA as well as several presecretory proteins (Müller et al., 2000; Linde et al., 2003).

Even though the general mechanisms of action and structures of DnaK, GroEL, and TF chaperones are widely conserved among organisms, their specific roles and their contributions to proteostasis maintenance differ between bacteria. A clear example of such divergence is displayed by the phenotypic differences between *E. coli* and *B. subtilis* chaperone-deficient mutants. Although in both organisms *groES* and *groEL* are essential genes at all temperatures (Fayet et al., 1989; Commichau et al., 2013), single and double deletions of *dnaK* and *tig* (encoding DnaK and TF) give different effects. In *E. coli*, DnaK is essential for growth at high or low temperature (Paek and Walker, 1987; Bukau and Walker, 1989), and it plays a crucial role under both optimal and proteotoxic stress conditions. Its absence causes cellular defects such as reduced growth rates, dysregulation of the heat-shock genes, and abnormal cell division (Paek and Walker, 1987; Bukau and Walker, 1989, 1990). DnaK is not only needed for the folding of a large number of *E. coli* “thermolabile” proteins (Mogk et al., 1999), it also regulates the heat shock sigma factor σ^32^ (Gamer et al., 1992; Liberek et al., 1992).

Deletion of *tig* in *E. coli* also leads to cellular defects: it reduces the cell’s tolerance to SDS/EDTA and vancomycin, thereby reducing outer membrane integrity (Oh et al., 2011), and induces the heat shock response (Deuerling et al., 2003). In *E. coli*, DnaK and TF possess overlapping functions in protein folding, and their shared role seems to be crucial for maintaining proteostasis, even under typical growth temperatures (Deuerling et al., 1999, 2003; Teter et al., 1999; Genevaux et al., 2004; Calloni et al., 2012). This redundancy might explain why a double deletion of *dnaK* and *tig* is synthetically lethal at temperatures above 30°C (Deuerling et al., 1999; Teter et al., 1999; Genevaux et al., 2004). Another example of chaperone collaboration is found between Hsp70 and the ATP-dependent Hsp90 chaperone, which in eukaryotes are well known to function together to orchestrate the proteostasis network (Schopf et al., 2017). In bacteria, the function of the Hsp90 homolog HtpG is not as well-characterized [see Wickner et al. (2021) for the latest review], but it has been shown that HtpG and DnaK systems also collaborate during the protein folding process in *E. coli* (Genest et al., 2011). DnaK-HtpG interaction involves the DnaJ-like protein CbpA (Genest et al., 2015). Unlike in eukaryotes, HtpG is not essential for the growth of many bacteria including *E. coli* and *B. subtilis*, and deletion of *htpG* causes minor growth defects after temperature upshifts in both organisms (Bardwell and Craig, 1988; Thomas and Baneyx, 1998; Versteeg et al., 1999). In addition, recent proteomic studies suggest that HtpG enhances degradation of DnaK/DnaJ client substrates (Fauvet et al., 2021).

While the implications and roles of chaperones in proteostasis are well-characterized in *E. coli*, far less is known for other bacteria including *B. subtilis*. In stark contrast to *E. coli*, the absence of DnaK, TF or both proteins in *B. subtilis* does not affect cell viability in the 16–52°C temperature range (Schulz et al., 1995; Gothel et al., 1998; Reyes and Yoshikawa, 2002). Apart from a very short study (Reyes and Yoshikawa, 2002), no characterization of the effects of a *dnaK tig* double deletion in *B. subtilis* has been published. *B. subtilis dnaK tig* double mutants are viable below 53°C, suggesting that folding of nascent peptide chains is assisted also by other proteins than DnaK and TF (Reyes and Yoshikawa, 2002).

*B. subtilis* DnaK and TF are subjected to regulation by phosphorylation. Tyrosine residue 601 in the C-terminal region of DnaK can be phosphorylated by the PtkA kinase and dephosphorylated by the PtpZ phosphatase, influencing its chaperone activity and survival of the cell upon heat-shock (Shi et al., 2016). In the case of TF, phosphorylation of Arg45 by the McsB kinase negatively influences its association with the ribosome (Zhou et al., 2019). Interestingly, spore germination requires Arg45 to be dephosphorylated by the YwlE phosphatase, since this licenses TF to interact with ribosomes and resume translation (Zhou et al., 2019). These observations are in line with the notion that chaperones possess host-specific roles, in addition to their conserved functions.

## Role of AAA+ Proteases, Adaptors, and Degradation Tags in Clearance of Aberrant Proteins

Unfinished, damaged, misfolded, or unneeded proteins are eliminated from the cell to maintain proteome integrity. In *B. subtilis*, degradation of most cytoplasmic proteins is performed by the conserved AAA+ family of intracellular proteases (AAA+; ATPases associated with a variety of cellular activities), which recognize, unfold, and degrade specific protein substrates (Sauer and Baker, 2011). *B. subtilis* has seven AAA+ proteases: ClpCP, ClpEP, ClpXP, ClpYQ, LonA, LonB, and FtsH (Elsholz et al., 2017), whose mechanisms of action have been described in numerous reviews (Sauer et al., 2004; Kirstein et al., 2009; Sauer and Baker, 2011; Olivares et al., 2016; Elsholz et al., 2017). The Clp complexes consist of an AAA+ unfoldase coupled to an ATP-dependent serine protease, whereas LonA, LonB, and FtsH have both unfoldase and protease domains within a single polypeptide (Elsholz et al., 2017).

Proteolysis can be regulated by adaptor proteins, which provide substrate specificity to proteases, usually by interacting with both substrate and protease. Several adaptor proteins have been characterized in *B. subtilis*, such as the ClpCP adaptor proteins MecA, YpbH, and McsB, and the ClpXP adaptor proteins YjbH and CmpA (Elsholz et al., 2017). The mechanism of adaptors often involves tethering the substrate to the protease to increase the local substrate concentration to facilitate proteolysis (Battesti and Gottesman, 2013). Less common mechanisms of adaptors are also known. For instance, the ClpXP adaptor protein YjbH does not appear to directly interact with ClpX (Chan et al., 2012), but enhances degradation of the stress-responsive regulator Spx by binding and stabilizing it, promoting its recognition by ClpXP (Awad et al., 2019).

AAA+ proteases or their respective adaptors recognize short degradation signals (degrons) located at the N-terminal, internal, or C-terminal position of protein substrates (Kirstein et al., 2009). A degron with relevance in protein homeostasis maintenance is the C-terminal SsrA degradation tag, which is added co-translationally by the transfer-messenger RNA (tmRNA) system to unfinished polypeptides when ribosomes stall (Keiler et al., 1996; Moore and Sauer, 2007). Truncated polypeptides challenge the stability of the proteome, and it is important that they are eliminated from the cell. SsrA-tagged polypeptides are typically degraded by the ClpXP protease complex (Sauer and Baker, 2011), although in *E. coli*, ClpAP and FtsH proteases can also recognize and degrade SsrA-tagged proteins (Gottesman et al., 1998; Herman et al., 1998). Cryo-EM studies have provided a detailed molecular mechanism of SsrA-tagged substrate recognition by ClpXP. Specific binding of the SsrA degron to ClpX triggers a ClpX conformational change from a “closed-pore” conformation to an “open-pore” conformation, allowing substrate translocation through the channel and subsequent non-specific interactions of the unfolded substrate with inner channel residues (Fei et al., 2020).

Interestingly, a novel ClpXP proteolytic mechanism for degradation of unfinished polypeptides in *B. subtilis* that is redundant with the SsrA tagging has been uncovered (Lytvynenko et al., 2019). Here, the *B. subtilis* RqcH recognizes stalled ribosomes and recruits tRNA*^Ala^* to mark aberrant nascent chains for degradation with C-terminal poly-alanine tails, which are recognized by ClpXP (Lytvynenko et al., 2019). Because the ALAA motif of the SsrA tag and poly-alanine tails are similar, it would be no surprise if their recognition and degradation mechanism would be similar. However, whether the poly-alanine tagged proteins are also degraded by ClpAP and FtsH in *B. subtilis* remains to be answered.

Another degradation tag of vast importance in *B. subtilis* proteostasis is the phospho-arginine (pArg) tag introduced by McsB (Trentini et al., 2016). McsB is conserved among Gram-positive bacteria and functions both as an adaptor protein for ClpCP (Kirstein et al., 2007) and as an arginine kinase with a major role in eliminating hundreds of damaged proteins from the cytoplasm, particularly under proteotoxic stress conditions (Elsholz et al., 2012; Trentini et al., 2016). McsB phosphorylates arginine residues, marking proteins for degradation by ClpCP (Trentini et al., 2016). Among the McsB phosphorylated proteins are the protein quality control members CtsR, HrcA, GroEL, TF, ClpC, and ClpP (Schmidt et al., 2014). In the case of the transcriptional repressors CtsR and HrcA, phosphorylation of residues in their DNA-binding domains greatly contributes to induction of the proteotoxic stress response (Kirstein et al., 2005; Fuhrmann et al., 2009; Schmidt et al., 2014). A recent study uncovered the molecular mechanism of McsB targeting (Hajdusits et al., 2021). McsB assemble into octamers, stabilized by auto-phosphorylation to form a molecular chamber-like structure, with the kinase active site buried inside (Hajdusits et al., 2021). McsB octamers are formed upon proteotoxic stress conditions, when McsB levels increase, and possess high selectivity for phosphorylation of unfolded proteins, which are able to access the kinase chamber through a narrow entrance. The phosphorylated proteins are thus targeted for degradation by ClpCP (Hajdusits et al., 2021).

Although it is likely that *B. subtilis* Lon proteins and FtsH play some role in protein quality control, experimental evidence is lacking. However, roles in degradation of regulatory proteins have been reported (Thi Nguyen and Schumann, 2012; Bradshaw and Losick, 2015; Mukherjee et al., 2015).

## Subcellular Localization of the Protein Quality Control Machinery

*B. subtilis* ClpC, ClpE, ClpX, and ClpP have been shown to co-localize with heat-induced protein aggregates or PorA inclusion bodies (Kruger et al., 2000; Jurgen et al., 2001; Miethke et al., 2006). In non-stressed cells, without aggregates, ClpP appears in the cytoplasm, while ClpC and ClpX are found both in the cytoplasm and associated with the membrane (Kruger et al., 2000; Jurgen et al., 2001). Three independent publications reported in 2008 that *B. subtilis* GFP-tagged Clp proteins, such as ClpX and ClpP, form foci with a cell polar localization pattern (Kain et al., 2008; Kirstein et al., 2008; Simmons et al., 2008). The subcellular localization of Lon seems to be developmentally regulated: *B. subtilis* LonA-GFP associates with the nucleoid under normal growth, and with the forespore during sporulation (Simmons et al., 2008).

It is important to note that used fluorescent protein tags lead to clustering artifacts when fused to homo-oligomers such as Clp proteins, and that, at least in *E. coli*, Clp proteins are homogenously distributed in the cell (Landgraf et al., 2012). Therefore, the native sub-cellular localization of the *B. subtilis* proteolytic machinery should be revaluated.

Localization of chaperones seems to be conditional to stress in several bacteria. For example, in *E. coli* the co-chaperone DnaJ mediates ATP-DnaK binding to protein aggregates (Acebron et al., 2008). Moreover, large heat-induced protein aggregates localize at the cell poles, and such a localization requires DnaK and DnaJ, as well as ATP synthesis and the membrane proton motive force (Rokney et al., 2009). DnaK and the ClpB disaggregase are essential for dissolving polar aggregates (Rokney et al., 2009), but it is not clear whether the polar localization of aggregates in *E. coli* is energy-dependent, since other studies claim it to be a passive process, driven by the molecular crowding in the nucleoid region (Winkler et al., 2010; Coquel et al., 2013; Gupta et al., 2014; Neeli-Venkata et al., 2016; Oliveira et al., 2016). Large, polar localized, protein aggregates are asymmetrically inherited in *E. coli*, as division generates cells with aggregates at the old cell poles (Lindner et al., 2008; Winkler et al., 2010). In *B. subtilis*, protein aggregates have also been shown to locate at cell poles (Kirstein et al., 2008; Runde et al., 2014; Stannek et al., 2014; Hantke et al., 2019; Schafer et al., 2019), but their inheritance after cell division has not been studied.

To our knowledge, chaperone localization in *B. subtilis* has been only addressed in few studies. *B. subtilis* GFP-DnaK localizes as multiple discrete foci proximal to the membrane (Meile et al., 2006). In response to short-term ethanol stress, phosphorylated DnaK and GroEL chaperones are recruited to the *B. subtilis* cytoplasmic membrane (Seydlova et al., 2012).

Collectively, it seems that to cope with proteotoxic stress the cell redirects the protein quality control machinery to sub-cellular areas, containing protein aggregates, such as the cell poles. Interestingly, protein aggregates are typically associated with detrimental effects for cellular fitness (Ross and Poirier, 2004; Lindner et al., 2008; Mortier et al., 2019), but their presence has been reported to pre-adapt lineages to subsequent proteotoxic stress (Govers et al., 2014; Mortier et al., 2019). Such pre-adaptation may arise from the increased levels of protein quality control agents such as proteases and chaperones that co-localize with protein aggregates (Kruger et al., 2000; Jurgen et al., 2001; Acebron et al., 2008; Govers et al., 2014; Mortier et al., 2019).

## Regulators of the Proteotoxic Stress Response

During proteotoxic stress, response mechanisms are activated which help the bacterium adapt to the new cellular or environmental condition. Of particular importance in *B. subtilis* are the HrcA and CtsR regulators. HrcA represses transcription of the *hrcA-grpE-dnaK-dnaJ-yqeT-yqeU-yqeV* and the *groES-groEL* operons (Schumann, 2016), and thus regulates the synthesis of chaperones. HrcA levels are depleted upon proteotoxic stress by a feedback mechanism involving the GroEL-GroES chaperone complex (Mogk et al., 1997; Schumann, 2016). CtsR, a regulator of protein degradation, represses transcription of the *ctsR-mcsA-mcsB-clpC-radA-disA* operon, and the *clpP* and *clpE* genes (Derre et al., 2000; Kruger et al., 2001; Elsholz et al., 2010). Regulation by CtsR involves a complex regulatory network, where McsB, McsA, and ClpCP play important roles in derepressing the CtsR regulon upon proteotoxic stress conditions [for a review, see Elsholz et al. (2017)].

Another player in the proteotoxic stress response is the Spx protein. Spx was initially characterized as a global regulator of the thiol-specific oxidative stress response (Nakano et al., 2003), controlling ∼144 transcriptional units (Rochat et al., 2012). However, an increasing number of studies have reported the involvement of Spx in the response to other stress conditions, such as heat shock and compounds targeting the cell wall (Runde et al., 2014; Rojas-Tapias and Helmann, 2018; Schafer and Turgay, 2019). The view that Spx is an important regulator of the proteotoxic stress response is becoming established (Rojas-Tapias and Helmann, 2019a,b; Schafer et al., 2019). Spx interacts with the C-terminal domain of the α-subunit (αCTD) of the RNA polymerase (RNAP), activating or repressing target genes in order to cope with the stress (Zuber, 2004; Newberry et al., 2005; Reyes and Zuber, 2008; Lamour et al., 2009; Nakano et al., 2010; Rochat et al., 2012). As revealed by structural studies, redox activated Spx with a disulfide bond between the two cysteine residues (Cys10 and Cys13) interacts both with the αCTD and σ*^A^* in the holo RNAP, and this complex binds to the −44 position of promoter DNA to enhance transcription activation (Shi et al., 2021). Among the Spx-induced genes are *trxA* (thioredoxin) and *trxB* (thioredoxin reductase), as well as the *clpX*, *clpE*, and *clpC* genes, and putatively *clpP* (Nakano et al., 2003; Rochat et al., 2012). Spx also induces the *ctsR* operon (Rojas-Tapias and Helmann, 2019a).

Control of the cellular level and activity of Spx involves many layers of regulation that are fine-tuned depending on the type of stress [reviewed in Rojas-Tapias and Helmann (2019b)]. The most important layer of Spx regulation seems to be through ClpXP proteolysis. Efficient degradation of Spx under normal conditions requires the ClpXP adaptor protein YjbH, which aggregates upon proteotoxic stress conditions and causes a decrease in Spx proteolysis (Nakano et al., 2003; Larsson et al., 2007; Garg et al., 2009; Engman and von Wachenfeldt, 2015). By hydrogen-deuterium exchange mass spectrometry it was determined that binding to YjbH decreases the Spx dynamics, reducing the conformational entropy and probably allowing a more efficient recognition of its C-terminal end, needed for ClpXP degradation (Awad et al., 2019). ClpCP and its adaptor McsB are also involved in Spx degradation, although to a lesser extent (Rojas-Tapias and Helmann, 2019a). Interestingly, the Spx paralog MgsR, which is also involved in the oxidative stress response (Reder et al., 2008), has been shown to interact with McsB upon ethanol stress, and McsB enhances MgsR degradation by ClpXP *in vivo* (Lilge et al., 2020).

Proteotoxic stress conditions also induce the general stress response, which is governed by the alternative sigma factor σ*^B^* and is one of the most important non-specific stress response mechanisms of *B. subtilis*. The σ*^B^* regulon is induced through a signal transduction cascade, involving the RsbV, RsbW, and RsbX regulators, and comprises about 200 genes, defined as class II heat-shock genes (Schumann, 2003; Nannapaneni et al., 2012). Other than heat, the general stress response is triggered by a wide range of stresses (Hecker et al., 2007). Genes included in the σ*^B^* regulon are, for example, genes that protect against elevated temperatures, such as *clpP* and *clpC*, and against oxidative stress, such as thioredoxin (*trxA*), peroxidase (*ohrA*), and superoxide dismutase (*sodA*) (Helmann et al., 2001; Petersohn et al., 2001; Price et al., 2001; Nannapaneni et al., 2012) and regulators (e.g., CtsR and Spx) (Hecker et al., 2007).

## Mechanisms to Downregulate the Translation Machinery During Proteotoxic Stress

Proteotoxic stress and other physiological demands on proteostasis may lead to insufficient protein folding capacity, resulting in accumulation of aberrant proteins. Reducing the rate of translation lowers the protein load, preventing further protein damage, and may help maintenance of proteostasis. Under proteotoxic stress conditions, such as heat or oxidative stress, *B. subtilis* downregulates transcription of translation-related genes, including ribosomal-protein encoding genes (*rplD*, *rpsC*, *rplW*, and *rpsJ*), and ribosomal RNA (rRNA) genes (Price et al., 2001; Leichert et al., 2003; Mostertz et al., 2004; Rochat et al., 2012; Schafer et al., 2019). In line with this notion, translation-related proteins have been found in protein aggregates in *E. coli* (Kwiatkowska et al., 2008; Dewachter et al., 2021). Moreover, the translation rate is reduced in *E. coli* cells containing a dysfunctional GroEL (Chapman et al., 2006), and protein folding is enhanced by slowing down translation rates in *E. coli* cells harboring mutant ribosomes (Siller et al., 2010).

Spx, apart from inducing transcription of stress-responsive genes, is also capable of repressing expression of genes for ribosomal proteins and rRNA (Nakano et al., 2003; Rochat et al., 2012; Schafer et al., 2019). Among the Spx downregulated genes are *rpoA* and *rpoC*, encoding for RNAP core subunits, and *lepA*, encoding for elongation factor 4 (EF4/LepA; Schafer et al., 2019), a paralog of the canonical elongation factor EF-G (Evans et al., 2008).

Downregulation of the translation machinery is also observed in cells lacking Spx (Schafer et al., 2019) suggesting that, at least under the stress conditions investigated (heat, oxidative, and cell wall stress), there are additional mechanisms to reduce translation in *B. subtilis*.

A downregulation of translation is frequently linked to the second messengers of nutrient starvation, ppGpp and pppGpp [collectively referred to as (p)ppGpp], which are synthesized by RelA (Rel in *B. subtilis*) and mediate the stringent response (Roghanian et al., 2021). Recently, a role of (p)ppGpp in slowing down the translation rate upon proteotoxic stress in *B. subtilis* has been suggested (Schafer et al., 2020).

McsB, which as mentioned before targets proteins for degradation by ClpCP, also has a potential role in regulating translation upon heat or oxidative stress, since it targets proteins related to translational control (Schmidt et al., 2014).

Inactivation of the *B. subtilis* methionine synthase MetE could also contribute to limit the translation rate by depleting the biosynthesis of the precursor of the initiation codon formyl-Met (Chi et al., 2011). Indeed, oxidative stress has been shown to inactivate *E. coli* MetE (Hondorp and Matthews, 2004), and oxidized MetE appears in the aggregate fraction of GroEL-defective mutants cells (Chapman et al., 2006). In response to the oxidative stress agents diamide or sodium hypochlorite, specific cysteine residues of *B. subtilis* MetE become either S-cysteinylated (Hochgrafe et al., 2007) or S-bacillithiolated, leading to its enzymatic inactivation (Chi et al., 2011). Moreover, MetE is among 108 identified S-thioallylated proteins caused by garlic sulfur compounds, and such compounds were found to induce the HrcA, CtsR, and Spx regulons (Chi et al., 2019).

## Concluding Remarks

*B. subtilis* is tolerant to drastic and rapid environmental changes by having a network of regulators and mechanisms controlling the synthesis, folding, post-translational modifications, sub-cellular localization and clearance of proteins to ensure proteostasis. Several of these processes are controlled differently in *E. coli* and *B. subtilis*, reflecting a long history of adaptations of the two model organisms to different niches.

## Author Contributions

Both authors listed have made a substantial, direct, and intellectual contribution to the work, and approved it for publication.

## Conflict of Interest

The authors declare that the research was conducted in the absence of any commercial or financial relationships that could be construed as a potential conflict of interest.

## Publisher’s Note

All claims expressed in this article are solely those of the authors and do not necessarily represent those of their affiliated organizations, or those of the publisher, the editors and the reviewers. Any product that may be evaluated in this article, or claim that may be made by its manufacturer, is not guaranteed or endorsed by the publisher.
